# Unusual Complication following a Myomectomy: Colic Migration of a Forgotten Abdominal Swab

**DOI:** 10.1155/2017/3962506

**Published:** 2017-01-26

**Authors:** Boyodi Tchangai, Fousseni Alassani, Mazamesso Tchaou

**Affiliations:** ^1^Department of Surgery, Teaching Hospital of Sylvanus Olympio, Lome, Togo; ^2^Department of Radiology, Teaching Hospital of Sylvanus Olympio, Lome, Togo

## Abstract

Surgical sponges are the most common retained foreign bodies following surgery. The morbidity of this condition is illustrated herein with the case of a 36-year-old female patient with a history of myomectomy 5 months before her admission into our unit for enterocutaneous fistula. Although imaging and etiological investigations were made, diagnosis was carried out only by laparotomy. The foreign body found was an abdominal swab that migrated from abdominal cavity to the colon causing several intestinal injuries. The lack of specific clinical signs and the death of the patient raise the necessity of preventing these complications that involve the surgeon liability.

## 1. Introduction

Surgical sponges are the most common retained foreign bodies following surgery [1]. They are known as textiloma or gossypiboma and occur with various surgical procedures including abdominal surgery. Textiloma results in an inflammatory reaction that becomes an encapsulated fibroblastic granuloma [2]. The clinical consequences are unpredictable and potentially disastrous. Enteric migration of abdominal textiloma is a rare complication that brings about considerable morbidity [1, 3]. We report a case of intracolic migration of an abdominal swab mistakenly left behind after myomectomy that resulted in death. The diagnosis difficulties and the importance of prevention are highlighted.

## 2. Case Description

A 36-year-old female patient was sent to our emergency unit for a subocclusive syndrome and enterocutaneous fistula. She has had a myomectomy in another health centre 6 months ago. On the 29th postoperative day, she experienced disorders with severe colicky abdominal pain and intermittent fever. The abdominal ultrasound was inconclusive and conservative management provided partial improvement. One week before her admission, the transit disorder got worsened with subocclusion.

**Figure 1:**
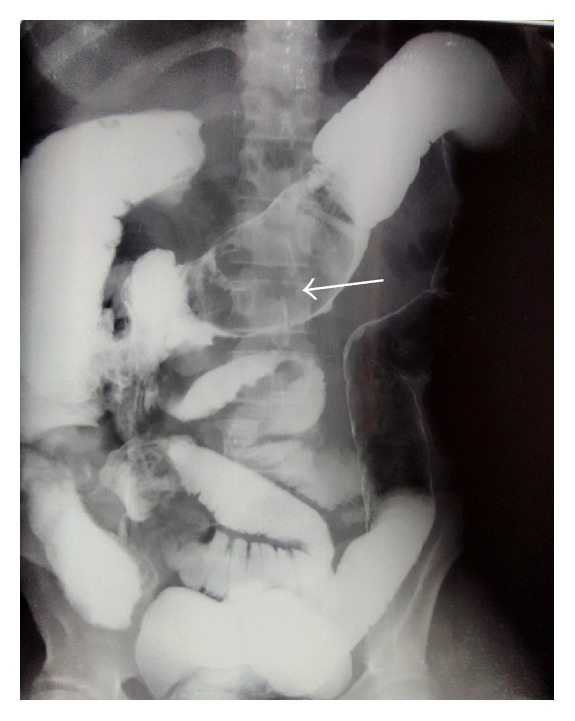
Gastrografin enema showing partially obstructing mass (textiloma) in transverse colon lumen with contrast leakage.

**Figure 2:**
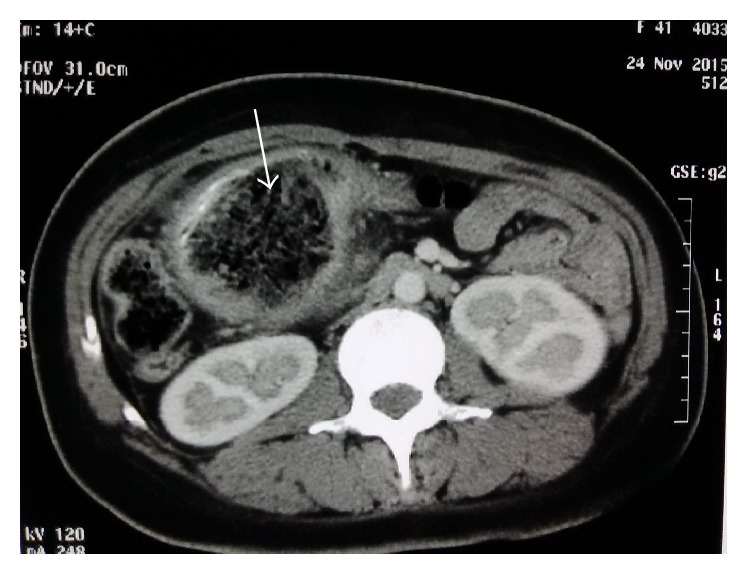
Injected abdomen CT scan showing limited spongiform mass with calcifications (textiloma) in the colon lumen.

**Figure 3:**
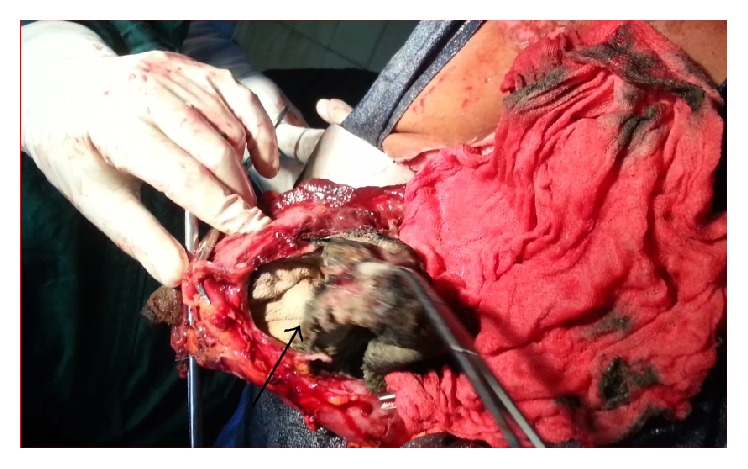
Image of perforated colon and abdominal swab in its lumen.

Initial tests revealed the following vital parameters: blood pressure 90/70 mmHg, pulse 110/min, respiratory rate 25/min, and temperature 39.1°C. Examination revealed a firm, mobile, and painful swelling in the epigastric area. There was tenderness in the right iliac fossa and enteric contents through a dehiscence of the laparotomy wound. Blood test revealed an anemia (Hb 8 gm/dL) and a high WBC count of 18000/mm^3^. Gastrografin enema showed a partially obstructing mass in the transverse colon and a fistula tract toward the right iliac fossa (Figure 1). Computed Tomography (CT) scan revealed low density, heterogeneous spongiform mass obstructing the colonic lumen (Figure 2). Emergent laparotomy showed multiple abscesses, and a tumor of the transverse colon. While performing colectomy we found a large abdominal swab (30/30 cm) that had migrated to the colon lumen (Figure 3). In addition, there was a complex enterocutaneous fistula involving the ileum and the ascending colon. The operation ended by colostomy and ileostomy. When reviewing the previous operation report, we found out that there had been a textile count procedure. The postoperative period was marked by a persistent fever and a resurgence of enterocutaneous fistula on the seventh day. The death occurred on the ninth day by septic shock.

## 3. Discussion

The retention foreign bodies cause many problems, both diagnostically and therapeutically. Textiles are the most frequent foreign bodies. Their frequency varies between 1/1000 and 1/10000, but this incidence is probably underestimated because of the medicolegal problems associated with these accidents reports [3]. Textile in the abdomen causes a foreign body reaction with exudation or encapsulating granuloma with intestinal adhesion [2]. Exudative reactions may be accompanied with secondary infection and early clinical manifestations while granulomatous reactions can remain asymptomatic for long periods of time. Transmural migration is a rare complication. Zantvoord et al. [4] found 64 cases in the international literature that were mainly located in the ileum between 1960 and 2007. A colic migration as we noticed is even rarer. It may be a secondary location from the ileum via internal fistula [5] or by intestinal peristalsis [6].

Migration occurs followed by an inflammatory necrosis of the intestinal wall and/or an excessive pressure exerted by granuloma around textiloma on the intestinal wall [3, 5]. No risk factor of transmural migration is identified in the literature. The risk is present from the 10th postoperative week and remains theoretically permanent and even increasing with time [4]. Complications associated with migration are occlusion and internal fistulas [3, 5, 7]. It is uncommon that enterocutaneous fistula follows textiloma transmural migration as we noticed [8]. We believe that the large size of the gauze swab found could be an impediment to a sealed transmural migration as it is used to happen. A timely diagnostic of the textiloma could have helped to avoid such a complication. Plain abdominal radiograph is useful if textiles are marked and not damaged [1]. In ultrasonography, textiloma is suggested by a hypoechoic tumor with a hyperechoic rim and posterior shadowing [7]. CT scan typically shows a well limited hypodense tumor or a spongiform aspect with heterogeneous density including calcifications and air bubbles [9]. Despite that fact, the differential diagnosis with fecal impaction, tumors, and postoperative abscesses can be a challenging situation [6].

The natural course of transmural migration is exceptionally good with the expulsion of textiloma by the anus [4]. In most cases, surgical intervention is required with a bowel resection or enterotomy according to the local conditions [5–8]. The postoperative course is usually good. The death in our case was related to the delay in diagnosis which led to poor local conditions, dissection difficulties, subsequent fistula, and uncontrolled sepsis. This poor outcome recalls the importance of prevention. It is interesting that the postoperative count of textile in our observation was declared complete as in 88% of textilomas cases [10]. This shows that negligence is not the only aspect of the issue but also prevention must take into account the risk of human error. There are many tools to minimize the risks: exclusive use of marked gauze swabs, systematic X-ray, radio frequency, and bar codes [1]. The use of these instruments is an additional cost; however, the huge legal fees associated with textilomas could be prevented. Where there is lack of resources, systematic textile count remains the only means. Efficient and simple protocols of textiles count must be put in place to increase its reliability.

## 4. Conclusion

Abdominal textilomas are responsible for significant morbidity that can lead to the death of patients. These situations involve the entire responsibility of the surgeon, so necessary measures ought to be taken to prevent it.
